# Supplementary figures and images for: Fatigue in incident peritoneal dialysis and mortality: A real-world side-by-side study in Brazil and the United States
Source: PLoS One. 2022 Jun 24;17(6):e0270214. doi: 10.1371/journal.pone.0270214 (PMC9231740; doi:10.1371/journal.pone.0270214)

Survival probability by Fatigue

Fatigue    +    +    +    +

<= 35    35-40    40-50    >50

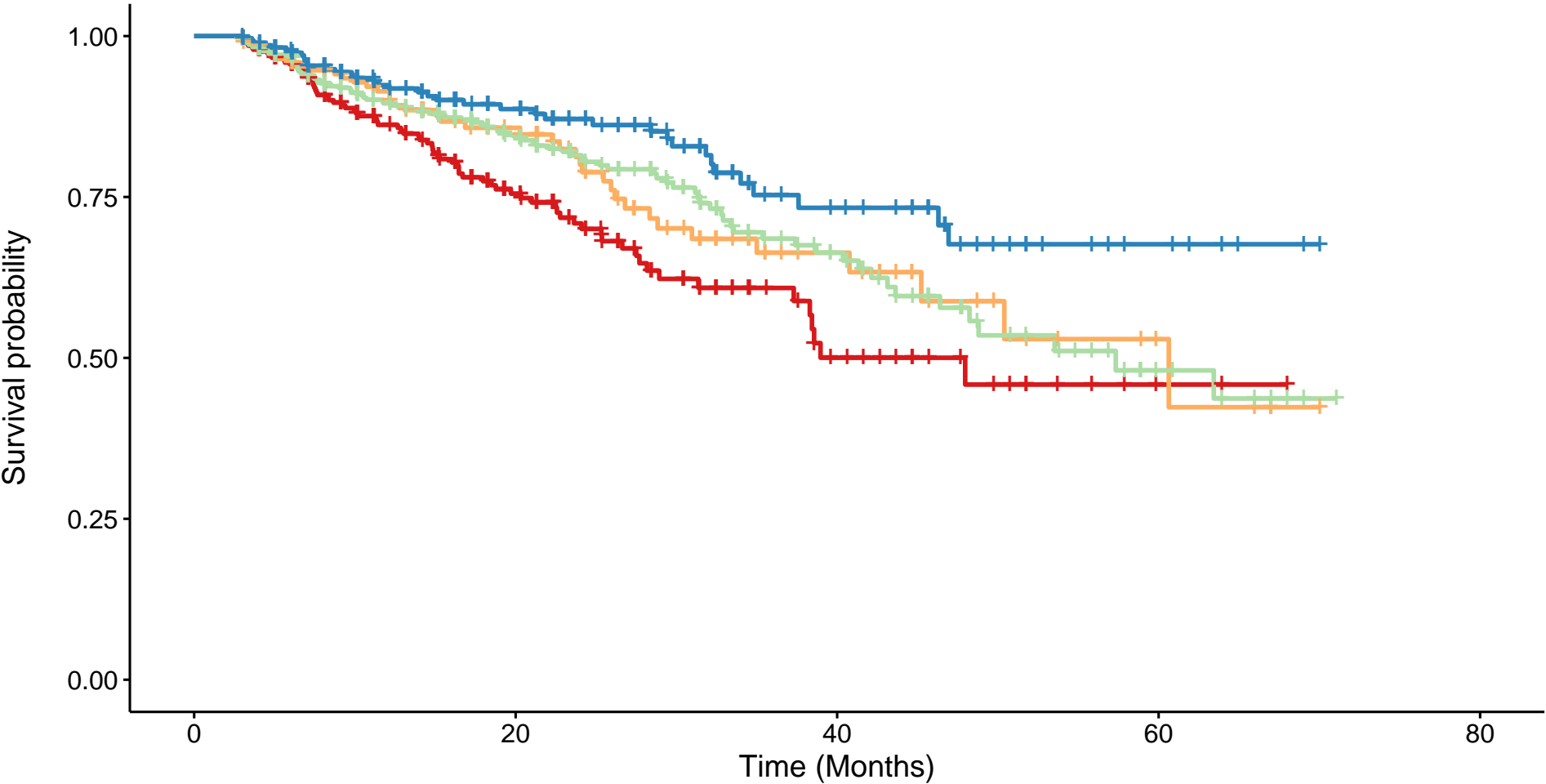

Number at risk

|         |       |     |     |    |    |   |
|---------|-------|-----|-----|----|----|---|
| Fatigue | <= 35 | 347 | 113 | 21 | 2  | 0 |
|         | 35-40 | 212 | 82  | 22 | 5  | 0 |
|         | 40-50 | 535 | 209 | 54 | 12 | 0 |
|         | >50   | 294 | 121 | 35 | 8  | 0 |

Time (Months)

Supplement: S1 Fig — (PDF) [file pone.0270214.s002.pdf]

# Survival probability by Fatigue

Fatigue + + + +

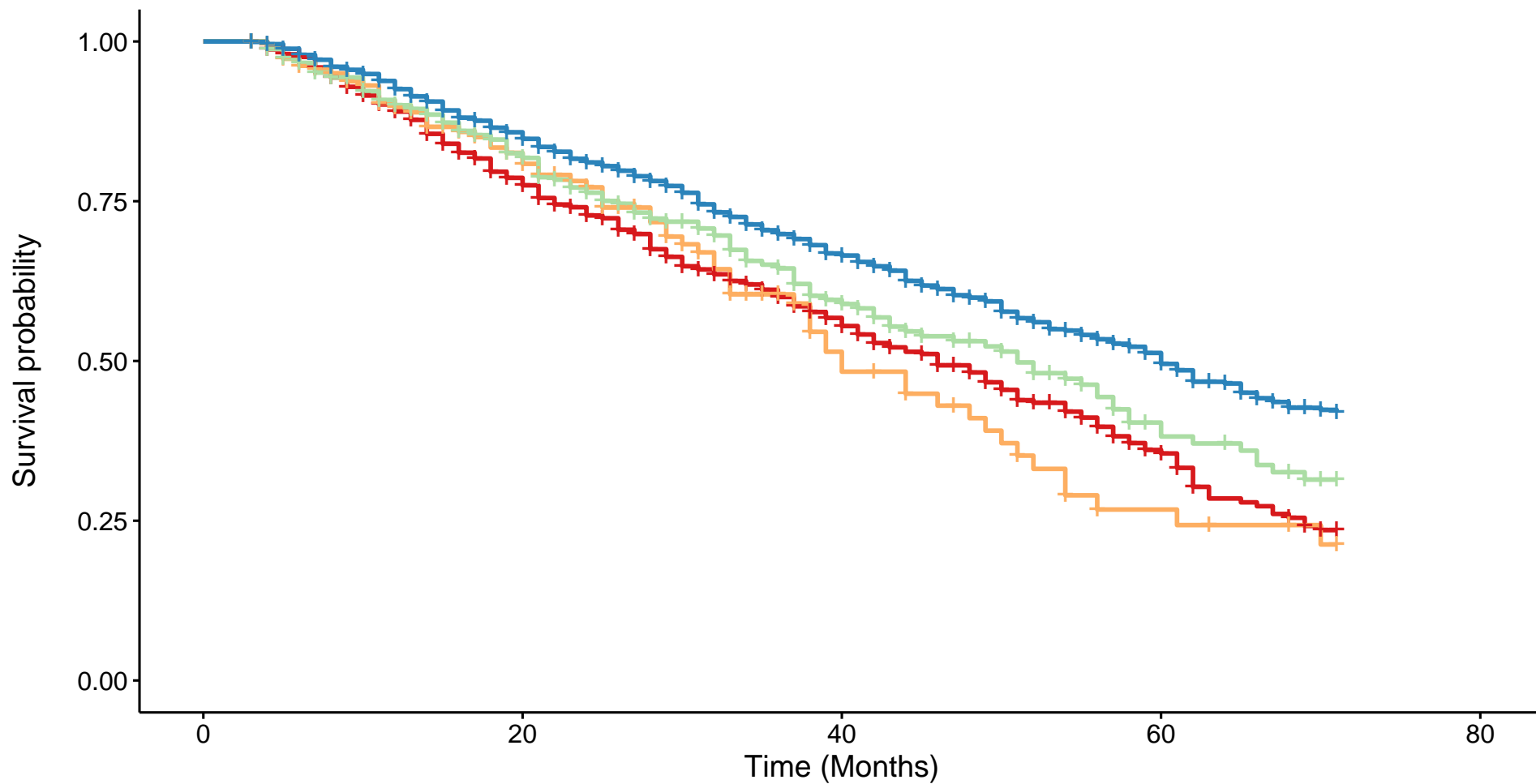

## Number at risk

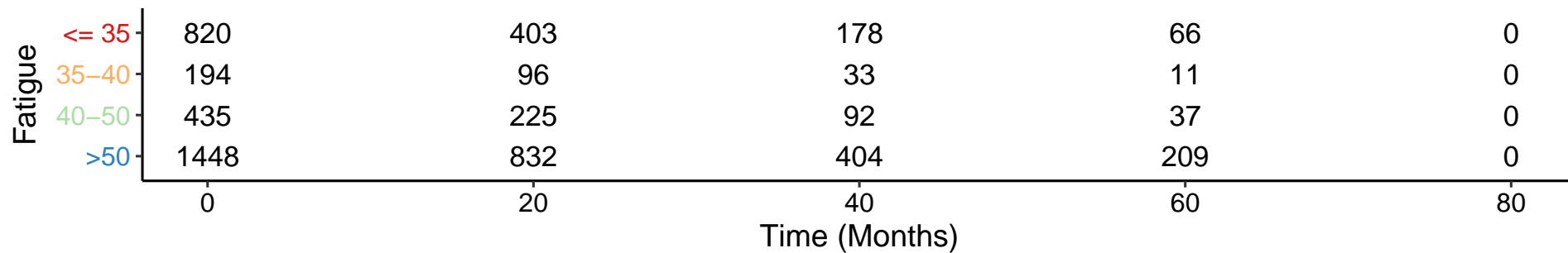

Supplement: S2 Fig — (PDF) [file pone.0270214.s003.pdf]
