# Supplementary material for: Fatigue in incident peritoneal dialysis and mortality: A real-world side-by-side study in Brazil and the United States
Source: PLoS One. 2022 Jun 24;17(6):e0270214. doi: 10.1371/journal.pone.0270214 (PMC9231740; doi:10.1371/journal.pone.0270214)
Supplement: Attachment — Submitted filename: PLOS_One_comments_20220302.docx [file pone.0270214.s004.docx]

Dear Dr. Dor

Please find below point-by-point answers to the excellent questions brought by the reviewers.

Reviewer #1: 1. The aim of this paper is to demonstrate that fatigue is related to mortality in the PD population. As this is true in the general population (fatigue being part of frailty), and has shown to be true in the HD population and as fatigue correlates with other markers of mortality, the authors should at least speculate why this may not be true for the PD population - and therefore that the study is worth carrying out. Was there ever any chance that this would not be true for the PD population - if not, is the study worth all the effort of carrying out?

**Response:** Thank you for this comment since it provides a great opportunity to expand the motivation for our work. Indeed, the association between fatigue and worse clinical outcomes seems to be very consistent across many disease states, including chronic kidney disease at its various stages, from non-dialysis to dialysis dependent. However, the potential causes and consequences of fatigue are varied and worth investigating, especially kidney failure populations. Specifically, transporting risk factors from the general population to kidney failure individuals is challenging, since many associations do not hold the latter group – the oftentimes called reverse epidemiology phenomena (doi: 10.1046/j.1523-1755.2003.00803.x.).

PD and HD patients are different in many ways – from demographics to clinical characteristics. Relevant to fatigue, cardiovascular risk factors and the incidence of heart failure are distinct in these two populations (doi: 10.1038/nrneph.2010.68.). Also, in PD, particularly at the incident period, fatigue can, as discussed in our paper, affect patients’ ability to undertake self-care, including catheter-related functions. Additionally, core outcomes, as ranked by patents, vary between HD and PD populations. In the latter, life participation and fatigue are highly important outcomes to be addressed in clinical research and practice, as highlighted by the SONG-PD initiative. Thus, understanding associations between fatigue and clinical outcomes remains as an important gap in PD literature. Characterizing outcomes associated to fatigue in PD can aid in the implementation of interventions to improve expected clinical and patient-reported outcomes.

Reflecting this great question, we further added the following in our introduction:

**Introduction, lines 64-69:** “*However, patients undergoing peritoneal dialysis (PD) are different compared to those in HD, for several reasons [11]. First, they tend to be younger and more functional. Second, the distribution of comorbidities is different between modalities. Therefore, fatigue causes, and associated factors, are often different between HD and PD. Finally, the transition period to dialysis initiation requires more active patient engagement in PD. Fatigue, then, can disproportionally pose higher risk for adverse outcomes in PD patients.”*

2. Following on from above, why the 2 populations. They are very different.

**Response:** Thanks for this point. We believe including two populations from large cohorts both in the US and in Brazil improve the external validity of our findings, since it increases representativeness of the findings even though there are distinctions in PD practice patterns and in patient populations in the world.

We added to following to clarify this point:

Discussion, lines 264-266. *The inclusion of cohorts with potentially distinct practice patterns may further add to the generalizability, since our associations were consistent in both datasets, despite the anticipated heterogeneities.*

3. Over what time period was the data collected?

**Response:** Thanks for the question. As stated in our methods, the period of data collection was December 2004 to January 2011.

4. It is stated that the observations are in incident patients - yet the BrazPD patients seem to have been on PD an average of >2years. So presumably BrazPD are prevalent and the US incident??

**Response:** Thanks for important question. All patients in the study are incident in PD, for both cohorts. Because patients may have been on HD before PD, the dialysis vintage may be longer than the period they are on PD. In BrazPD, a greater proportion of patients were on HD before transitioning to PD representing the distinct practice patterns between Brazil and the US. Importantly, this definition of incident PD patients is consistent across many registries and cohorts.

5. I am not a statistician

–

Reviewer #2: excellent paper – very interesting and well analysed

Thank you for your kind appreciation.

I wondered whether might be a sensitivity analysis around patients who did not complete the vitality scores – ie were there outcomes broadly similar to those who did. The reason is that the number who did complete the vitality score is a rather small number compared to the total group.

**Response:** Thanks for this comment, which is very important. We unfortunately are not able to pursue this sensitivity analysis due to the cohort definition used in vitality measurement in the protocol for the North America cohort.

However, our mortality estimates for the North America cohort is consistent to the USRDS reports (converted to 1000 patient-years basis), as seen in the figure below:

For the BrazPD, the mortality proportion reported in our paper is consistent to the one reported for the full cohort (doi: 10.3747/pdi.2013.00282) – 20% at our sample and 19% for the general cohort.

We added the following limitation:

Discussion, lines 259-262: *“Also, due to the study design, we included only patients who completed vitality scores within 90 days from PD start. This resulted in an exclusion of a relevant proportion of our sample. This may have resulted in selection bias, because patients who completed vitality scores may be different from those who did it.”*

I note the numbers of events in the supplementary Kaplan Meier curves are rather small and wondered what the explanation for this is.

Thanks for this observation. Indeed, in BrazPD, the number of events is lower than in the US cohort. We addressed these distinctions in our discussion. Regardless, the event rates reported in BrazPD are consistent with other PD cohorts (such as in DOI: <https://doi.org/10.1681/ASN.2007111188>).

Do you have data on the repeatability of the vitality scores - it would be imporant to know

**Response:** Again, this is a very insightful comment. Thanks. We do have longitudinal vitality scores data, but we feel that modelling it as time-varying exposure is an effort for a separate paper.

We included the following as a limitation:

Discussion, line 262-264: *Finally, we evaluated fatigue only at the period within 90 days from PD start. As fatigue is a dynamic exposure which may vary over time, a design evaluating longitudinal fatigue may be informative and should be considered in independent efforts*.
